# A Blast From the Past: Radiation Therapy During Childhood Causing Cardiac Fibrosis and Calcification Leading to Complete Heart Block

**DOI:** 10.7759/cureus.10709

**Published:** 2020-09-29

**Authors:** Yury Malyshev, Nnamdi Chukwuka, Arsalan Talib Hashmi, Sarah Rosanel, Guy Kulbak

**Affiliations:** 1 Cardiology, Maimonides Medical Center, Brooklyn, USA; 2 Medicine, Maimonides Medical Center, Brooklyn, USA; 3 Cardiology, Lahey Hospital & Medical Center, Burlington, USA

**Keywords:** radiation, radiation heart disease, complete heart block, rbbb, mri cardiac, cardiomyopathy

## Abstract

Complete heart block (CHB) in a young patient is a rare phenomenon necessitating an extensive workup to identify the etiology of conduction disturbance. Radiotherapy of the thorax is a known risk factor for cardiomyopathy; however, CHB is a rare complication. Here we present a case of a 46-year-old man who presented with CHB and was found to have significant cardiac fibrosis and calcification of the mitral valve annulus. His management required a multidisciplinary and multimodality approach to be able to identify childhood radiation as the cause of cardiomyopathy and establish a personalized management strategy with cardiac resynchronization therapy defibrillator.

This case highlights radiation therapy as an important cause of cardiac conduction abnormalities even decades later, and the importance of extensive search for other reversible etiologies using the multimodality approach.

## Introduction

Radiation-induced heart disease (RIHD) comprises a constellation of conditions that occurs as a result of exposure of the heart to radiation [1]. Exposure usually occurs during the treatment of childhood malignancies such as lymphoma, breast cancer, and teratomas in the anterior mediastinum.

Despite interventions taken to reduce the amount of radiation the heart receives during radiotherapy, RIHD still remains a condition that is fairly common. Unfortunately, recognition of RIHD is usually difficult to make because it manifests 20-30 years after the initial exposure. Here, we report a case of a 46-year-old man with a complete heart block (CHB) and nonischemic cardiomyopathy following a remote history of radiotherapy.

## Case presentation

**Figure 1:**
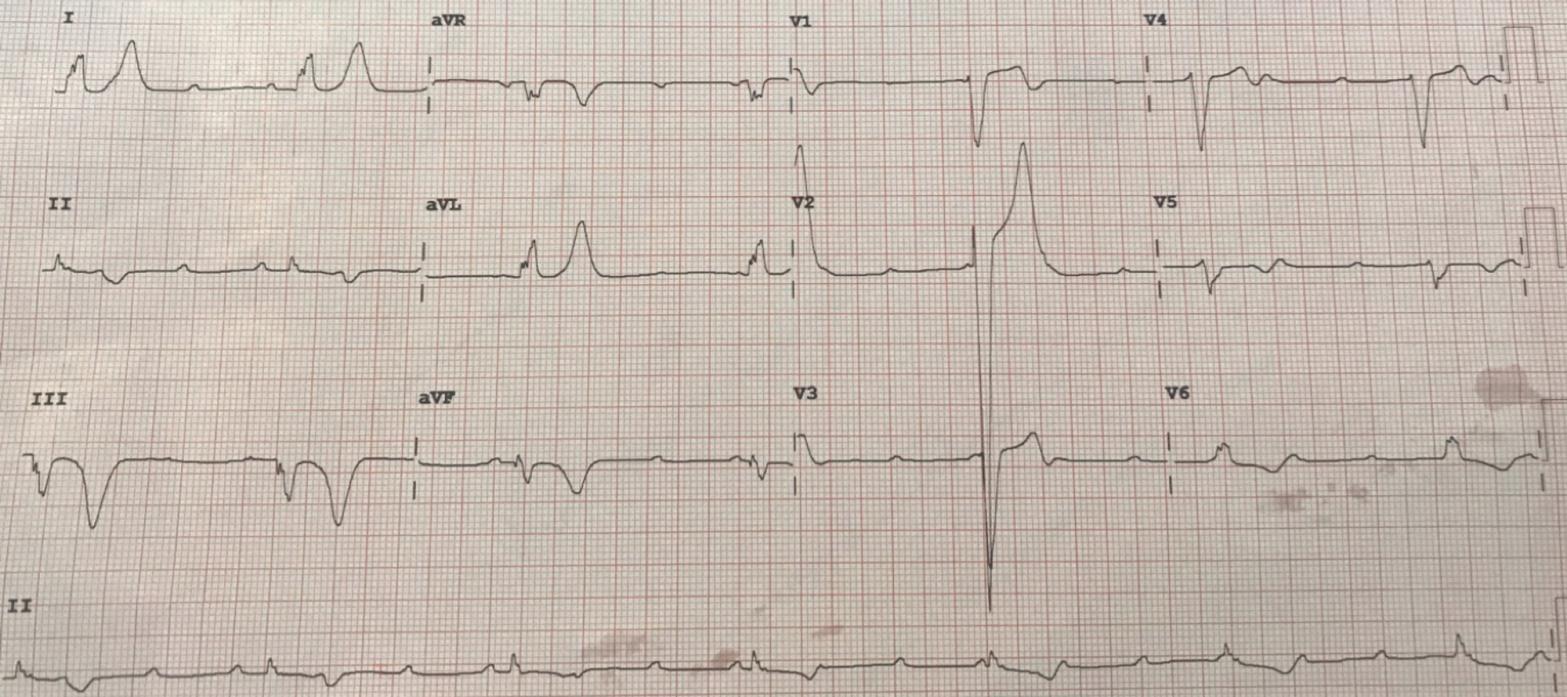
Electrocardiogram showing complete heart block

A 46-year-old man with no known significant comorbidities presented to the emergency department with complaints of episodic lightheadedness, nausea, and palpitations. He was sitting at a meeting, when suddenly he felt palpitations and dizziness. He reported a syncopal episode with similar preceding symptoms about one month ago. Workup with electrocardiogram (EKG) and stress test did not reveal obvious abnormality a month ago. On arrival to the emergency room (ER), he was bradycardic with mild shortness of breath. EKG in ER showed CHB (Figure 1).

**Figure 2:**
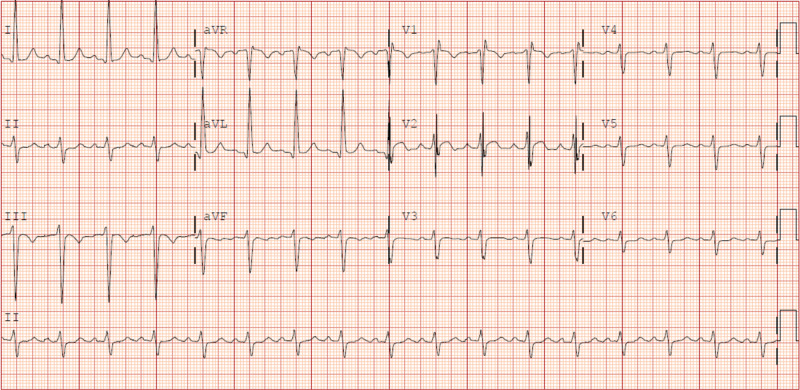
Electrocardiogram showing sinus tachycardia with right bundle branch block

Twenty minutes later without any interventions, EKG showed sinus tachycardia with right bundle branch block (RBBB) (Figure 2).

**Figure 3:**
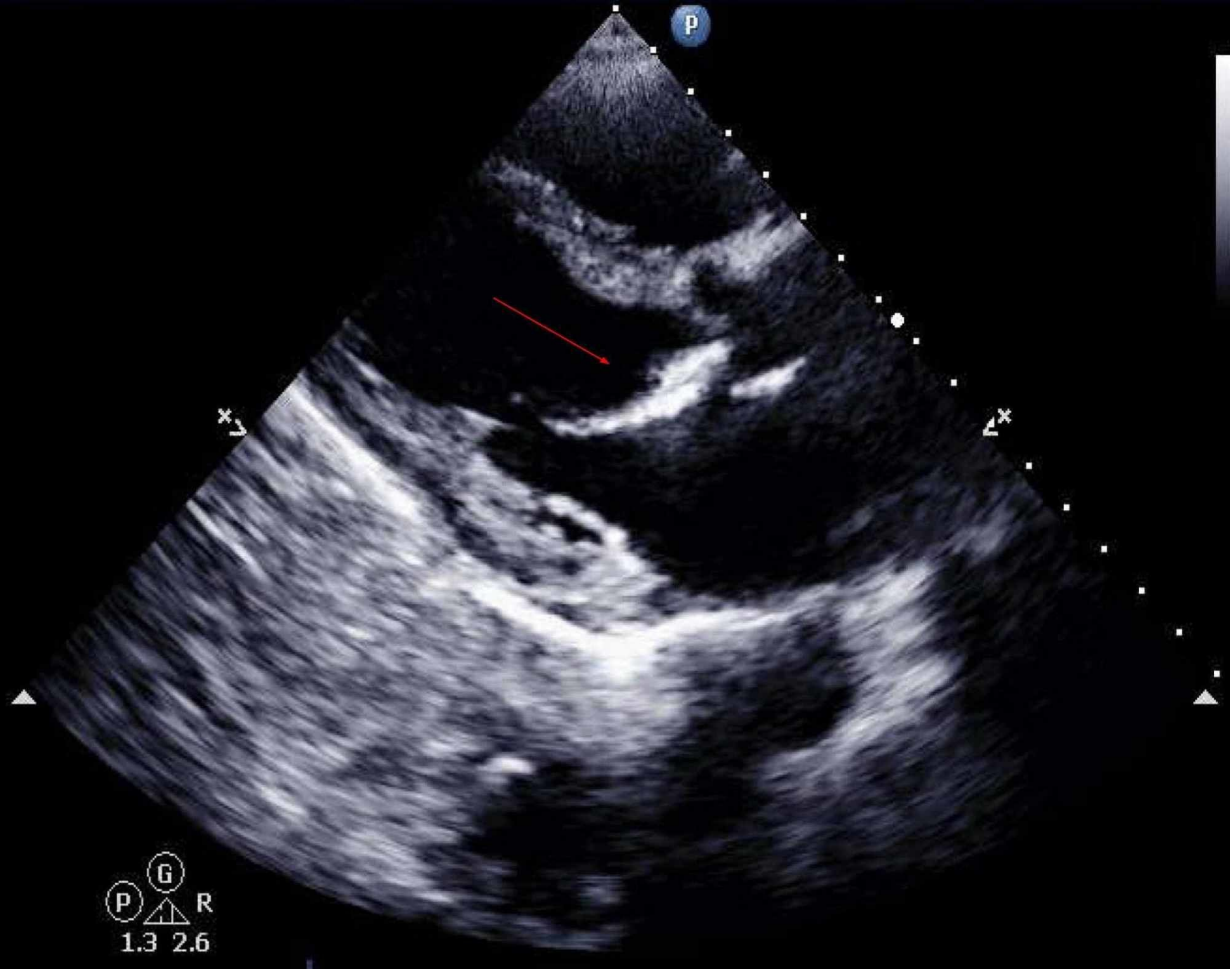
Transthoracic echocardiogram (long axis view) showing calcified anterior mitral leaflet The red arrow showing calcified anterior mitral valve leaflet

Echocardiogram showed left ventricular ejection fraction of 40%, abnormal septal motion, restrictive filling, dilated and hypokinetic RV, calcification of the anterior mitral valve and annulus of the mitral valve (Figure 3).

Coronary angiogram did not reveal coronary disease or calcification of the coronary vessels. During patients' hospital stay, telemetry monitoring did not show other conduction abnormalities. Upon further questioning, the patient admitted to having lymphoma 36 years ago treated with radiation to the thorax; he also regularly hunted in the woods in New York State.

**Figure 4:**
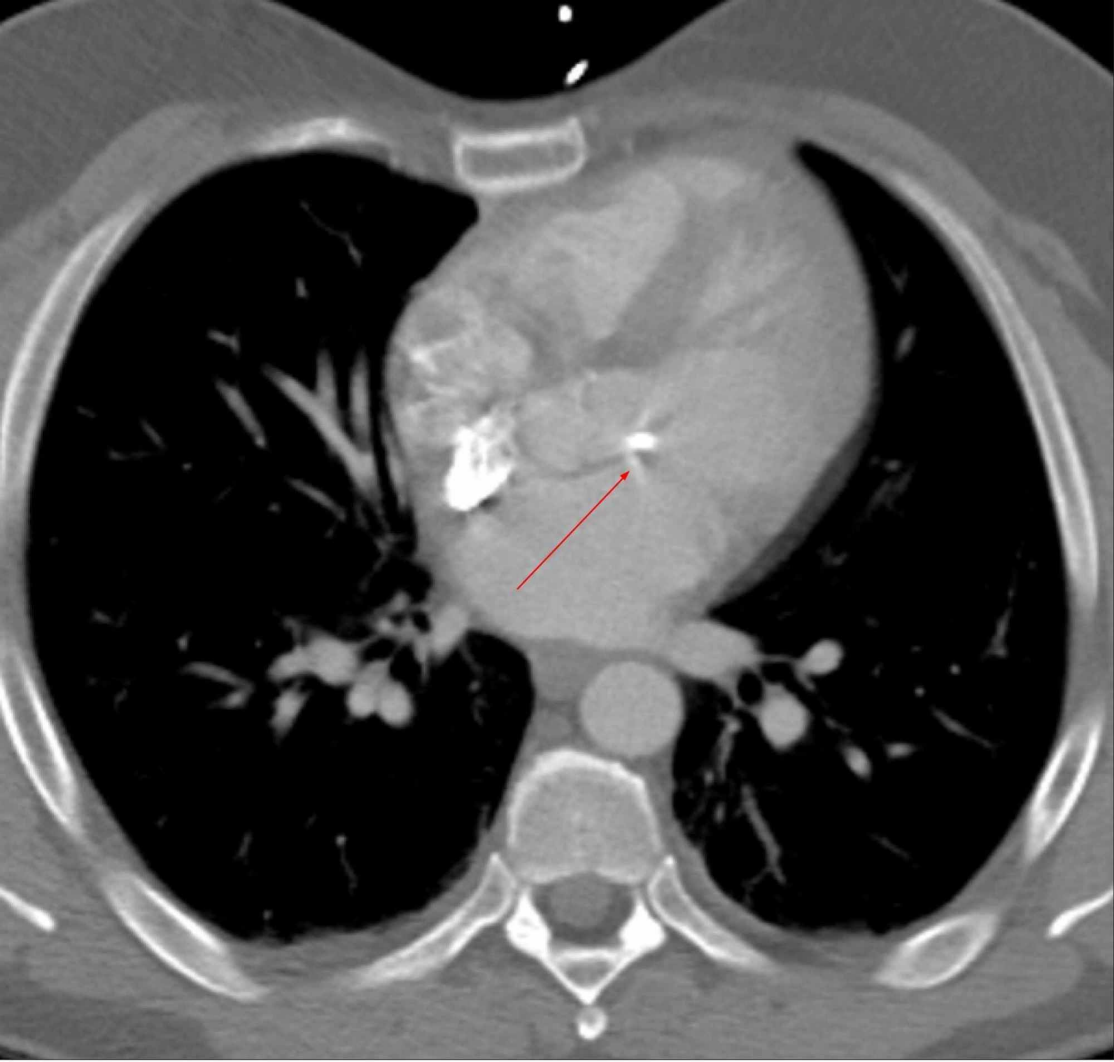
CT of the chest showing calcification of the mitral valve and no pulmonary pathology

Based on history and workup, the patient was suspected to have CHB secondary to Lyme disease, sarcoidosis, or radiation. Lyme titers, erythrocyte sedimentation rate (ESR), C-reactive protein (CRP), and angiotensin-converting enzyme were normal. CT chest did not identify lung pathology, but showed mitral valve calcification (Figure 4).

**Figure 5:**
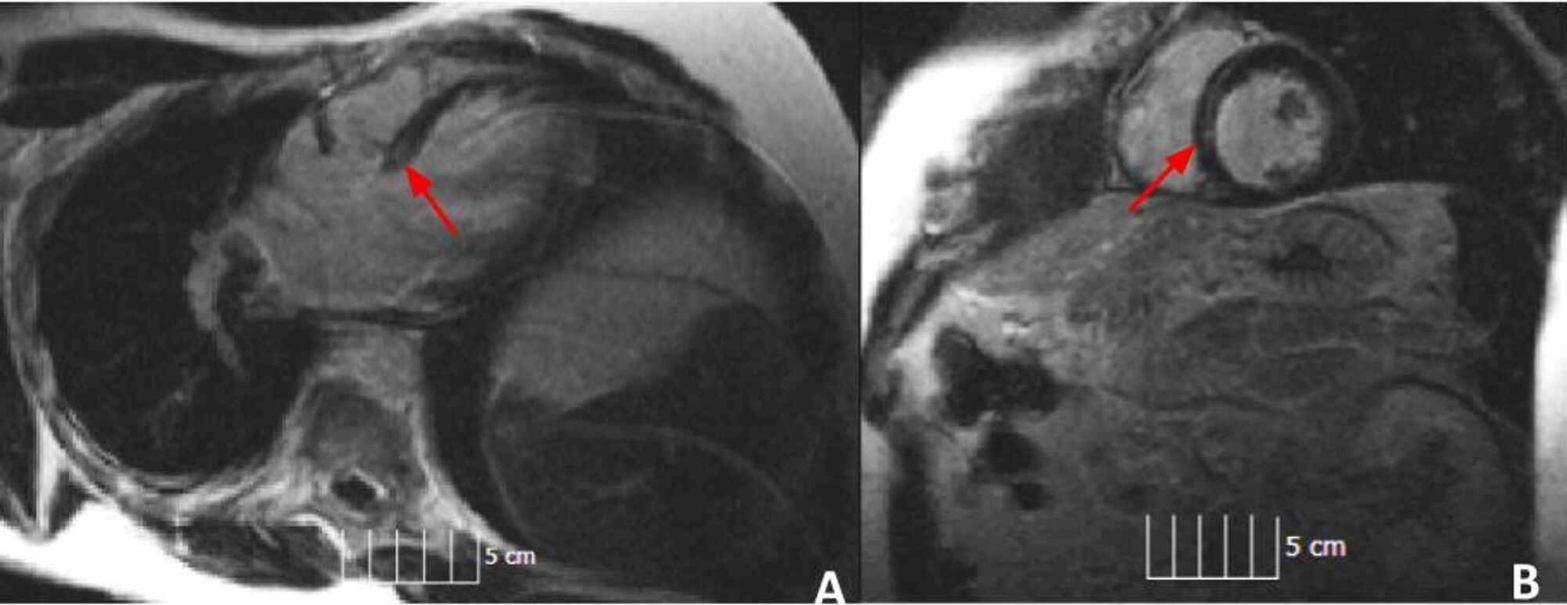
MRI of the heart showing fibrosis (red arrows) (A) Four chamber view. (B) Short axis view.

Cardiac MRI showed linear mid myocardial delayed enhancement in the anterior basilar septum consistent with sarcoidosis vs fibrosis (Figure 5).

Because Lyme disease and sarcoidosis workup has been negative, it was determined that childhood exposure to radiation is the etiology of cardiomyopathy, calcification, and conduction disturbance. Due to highly expected progression of patient's conduction abnormality, nonischemic cardiomyopathy without reversible causes, mid myocardial delayed enhancement, wide QRS (134 milliseconds), and a transient symptomatic CHB, a decision was made to implant not just a permanent pacemaker, but a cardiac resynchronization therapy defibrillator (CRT-D) to decrease the number of necessary procedures to upgrade devices in the future. 

## Discussion

Pathogenesis of RIHD is based on a number of mechanisms. These include generation of oxygen free radicals with oxidative stress, endothelial cell injury and inflammation, and apoptosis [1].

Conduction defects caused by RIHD consists of RBBB and left bundle branch block, CHB, QTc prolongation, and supraventricular and ventricular arrhythmias [2]. RBBB is the most common conduction defect due to the fact that the right bundle is the most anterior part of the cardiac conduction system [3]. Conduction defects due to RIHD often occur within two months to years after radiotherapy [4].

Studies have shown that approximately 74.5% of patients exposed to radiotherapy for Hodgkin’s disease develop a conduction abnormality [4]. Such a high number raise the necessity of screening these patients for postradiation conduction abnormalities.

Recognition of conduction defects caused by RIHD requires supportive history, an EKG, and a high index of suspicion. It is also important to rule out other causes such as coronary artery disease, Lyme disease, Chagas disease, and infiltrative diseases like amyloidosis, hemochromatosis, and sarcoidosis [5].

Prevention remains the bedrock for managing RIHD. Limiting the amount of radiation the heart receives has significantly reduced the incidence of RIHD. In addition, medications such as statins and angiotensin-converting enzymes inhibitors have been shown to reduce the effect of radiation on the heart [1]. 

There is no known cure for conduction defects caused by RIHD. Some studies showed that 70% of EKG abnormalities can spontaneously disappear within six months of radiotherapy [4]. However, if they persist, treatment is aimed at correcting the arrhythmia. As in the index case, radiation-induced CHB had no cure. Since the patient's radiotherapy had occurred many years ago, recovery was unlikely. Placement of a permanent pacemaker was the only option to control his conduction abnormality. However, the patient was also found to have mid myocardial late enhancement, which is independently predictive of sudden cardiac death [6]. Considering the extent of his disease was likely to progress with worsening of systolic function and risk of sudden cardiac death, a decision was made to place a CRT-D device to protect patient and decrease number of future procedures when device upgrade would be needed.

## Conclusions

RIHD remains a disease of exclusion. Suspicion should be always high when a relatively young patient presents with CHB in the appropriate clinical setting. However, other reversible causes of CHB should always be ruled out. 

As evidenced by our case and previous studies, the rate of postradiation conduction abnormalities is high. In our opinion, patients who were treated with radiotherapy to the mediastinum should be screened for postradiation abnormalities to identify first signs of conduction defects and avoid potential complication of CHB and other arrhythmias.
